# Combined Donepezil and Ethanolic Extract of Propolis Improved Memory Better Than Donepezil and Propolis Monotherapy in Wild Type* Drosophila melanogaster*

**DOI:** 10.1155/2018/3717328

**Published:** 2018-08-12

**Authors:** Emmanuel Tiyo Ayikobua, Ibrahim Semuyaba, Daniel Ejike Eze, Muhamudu Kalange, Mariam Nansunga, Alfred Omachonu Okpanachi, Abass Alao Safiriyu

**Affiliations:** ^1^Department of Physiology, Faculty of Biomedical Sciences, Kampala International University, Western Campus, P.O. Box 71, Bushenyi, Uganda; ^2^Institute of Biomedical Research Laboratory, Kampala International University, Western Campus, P.O. Box 71, Bushenyi, Uganda; ^3^Department of Physiology, Faculty of Biomedical Sciences, St. Augustine International University, P.O. Box 88, Kampala, Uganda

## Abstract

**Background:**

Donepezil is the most common drug used in the treatment of disorders associated with memory loss, especially that in Alzheimer's disease. Healthy individuals however have continued to use it as a memory enhancer. This study was aimed at evaluating the combined therapy of donepezil and propolis on cognition in* Drosophila melanogaster*.* Method. Drosophila melanogaster *flies were divided into five groups and fed with the different treatment doses of ethanolic extract of propolis and donepezil as follows: normal food, propolis 250 mg/mL, propolis 50 mg/mL, donepezil 0.001M, and donepezil 0.001M/propolis 50 mg/mL added to their food. The flies were fed from larval stage for 30 days. The memory and learning tests were conducted after every 10 days to assess improvement with time.

**Results:**

The results obtained showed that the combination of propolis with donepezil caused a remarkable improvement in both the short- and long-term memory. In addition, there was a dose dependent improvement with the administration of propolis.

**Conclusion:**

Propolis extract obtained from different parts of Uganda expressed cognitive improvement when coadministered with donepezil in wild type* Drosophila melanogaster*.

## 1. Introduction

The financial cost of caring for people with deteriorating memory is over £26 billion per year, including healthcare, social care, and unpaid care from family members [1]. This is more than the cost of cancer or heart disease [2, 3].

The increasing awareness of the general population on the cognition disorders and their burden to the society has led them to seek psychopharmaceuticals interventions to improve their cognitive function [4, 5]. The most commonly prescribed are the acetylcholinesterase inhibitors (AChEIs) such as donepezil, rivastigmine, and galantamine [5].

Donepezil is most associated with the treatment of Alzheimer's disease (AD). Despite the fact that it is effective in treating mild to moderate AD, there is limited data on either the cognitive or neural impact when administered to healthy older individuals [6]. Despite the indication of donepezil in Alzheimer's disease to improve memory, individuals have continued to use it in nondisease cases as a memory enhancer [7]. However, a study showed that the use of donepezil in the normal elderly population caused a negative effect on cognition [8].

Memory by definition is the internal records that organisms maintain that give them access to their past complete with all the facts they know and skills they have attained [8]. Memory is subdivided into short- and long-term memory. Short-term memory is the ability to recollect things that happened immediately up to a few days in humans. Long-term memory stores potentially much larger quantities of potentially unlimited duration and its capacity is immeasurable and encodes information semantically [8]. Learning is the process of modifying information already stored based on new input and experiences since memory is a subset of learning, and the first step in learning is memory [9].

The new cases reported with cognitive impairment rise with age, with about 5% of the population between 71 and 79 years old showing dementia [10, 11]. This rises to 37.4% of the 90 year olds and above globally. With regard to these incidence rates, the prevalence of poor memory and cognitive disorders also increases with increasing age rising to 43% for those between 65 and 75 years old, 51% for those 75-84 years old, and 88% for those 85 years old or older [12]. The fraction of people over 70 is projected to rise dramatically in the coming years with the globally increasing average life expectancy which has risen from 65 years 10 years ago to 72.5 and this is expected to rise to 90 years in the next 10 years due to the increasing health care services globally [13].

Mild cognitive impairment (MCI) describes a set of symptoms that involves various problems with cognitive functioning including memory loss, and problems with language, attention, and problem-solving which can be a normal part of the aging process. MCI later develops into dementia, including Alzheimer's disease, vascular dementia, frontotemporal dementia, and dementia with Lewy bodies, all of which are progressive, with people living for up to 12 years after initial diagnosis. Around 10 to 15% of people who experience MCI with memory loss will go on to develop dementia, most often Alzheimer's disease [14].

Propolis is a bee resinous product obtained from different parts of plants such as buds, bark, and tree exudes by bees [15]. Its chemical composition depends on its origin and varies vastly. Propolis has attracted much attention in recent years as a useful substance for medicines and cosmetics, although it has been used in folk medicines since the ancient years [16]. Propolis around the world is composed of estimates of about 50% resin and vegetable balsam, 30% wax, 10% essential and aromatic oils, 5% pollen, and 5% other substances. In tropical region propolis, especially east African propolis, the dominating chemical components are prenylated, phenylpropanoids (e.g., artepillin C), and diterpenes [17]. Diterpenes from Rosmarinus officinalis has been shown to have therapeutic potential for Alzheimer's disease [18].

Propolis was used in this study because it is rich in polyphenols which have been shown to have neuroprotective activity because of their ability to cross the blood brain barrier and exert their effects which include protection of neuronal cells from oxidative stress and neuronal inflammation associated with normal aging and chronic age related diseases [19].

Propolis having a broad spectrum of activities including antioxidant, anti-inflammatory, and neuroprotective functions and according to studies, few side effects and interactions make it a very useful remedy in management of cognitive disorders and mental disabilities [20].

*Drosophila* as a model organism for experimental studies has been chosen as one of the best models to study aging and age related diseases. It is inexpensive and easy to maintain in the laboratory, it can give rise to a large number of genetically identical progeny, it has a rather short lifespan of about 40-120 days depending on the diet and stress, and it shows complex behavior that includes learning and memory driven by a sophisticated brain [21]. The fly provides a very powerful genetic model for the analysis of brain and behavioral disorders related to human diseases. Its brain is complex enough to make to make fly behavior highly interesting and relevant to humans though it is very small for an in-depth structural and functional analysis [22]. Therefore, we investigated the role that combined and monotherapy of donepezil and extract of propolis would have in learning and memory of wild type* Drosophila melanogaster*. Our findings revealed that while propolis and donepezil monotherapy enhanced cognition in* Drosophila melanogaste*r, greater improvement was obtained when the two were coadministered.

## 2. Materials and Methods

### 2.1. Drosophila Stocks

For the experimentations, wild type (WT) Oregon-R-C mutant* Drosophila melanogaster *(Dm) flies were obtained from the Bloomington Stock Center and flown to Entebbe international airport, Uganda.

### 2.2. Drosophila Culture and Crosses

Flies were obtained from the Institute of Biomedical Research laboratory Kampala International University Western Campus, Uganda. These flies were then reared on standard commercial food for multiplication. The flies were then placed in vials containing the different treatments ranging from fresh normal food to that including propolis and donepezil (Table 1). They were left for 3 days to lay eggs and removed. The resulting larvae were left to develop, and the counting of days commenced.

### 2.3. Drosophila Grouping

The grouping was done as shown in Table 1.

### 2.4. Memory Assay

The memory tests were carried out at days 10, 20, and 30 following initiation of the experiment. In this test, two long vails are connected together using masking tape and one side of the vail is made dark (Dark chamber), while the other side is left transparent (light chamber) to allow the monitoring of fly behavior. The flies of different groups were first trained by assessing their ability for positive phototaxis. During this training, the flies were introduced through the open end of the tube with no light then the open end is closed with cotton wool. The flies were then allowed to exhibit positive photo taxis by moving towards the light chamber. Flies that moved to the light for every at least 8 of the 10 trials (80% pass) within 30 seconds were qualified for use.

Flies that were qualified for use were then subjected to the learning test.

Learning ability/training was assessed by placing a filter paper containing quinine at the end of the light chamber.

Acting as an aversive stimulus, the flies were then trained to associate the aversive stimulus with light side of the chamber by doing repeated trials allowing the flies to move towards the light chamber with quinine in each trial in a time of 30 seconds. Flies that learned and associated the light with the aversive stimulus avoided the light chamber on subsequent trials. This avoidance was recorded as a percentage calculated as follows:

((Number of times the fly avoids light chamber/number of trials)*∗*100)

Flies that exhibited 80% pass were then used for the short-term and long-term memory test as follows. Flies that learned were stored for 30 mins after which the test steps above were repeated and the percentage pass was calculated using the formula above. This was done for short-term memory. Long-term memory was assayed after 6 hours of flu resting. The average percentage pass was calculated and this was used in the statistical analysis.

### 2.5. Statistical Analysis

Statistical analysis was performed using Graphpad prism Version 7. Data was expressed as the mean percentage ± SEM. Data was analysed using two-way ANOVA and was followed by Bonferroni post hoc test for multiple comparison of monotherapy and combined treatment groups. P values were obtained and statistically significant differences were accepted at p < 0.05.

## 3. Results

The findings of this study revealed an improving trend in the use of donepezil 0.001M/propolis 50 mg/ml over time. The improvement over the days in short-term memory recorded was statistically significant following an ANOVA.

Donepezil showed positive results the first 20 days; however, on the 30th day there was a drop in short-term memory by a mean of 3.00% which however was not statistically significant.

**Figure 1:**
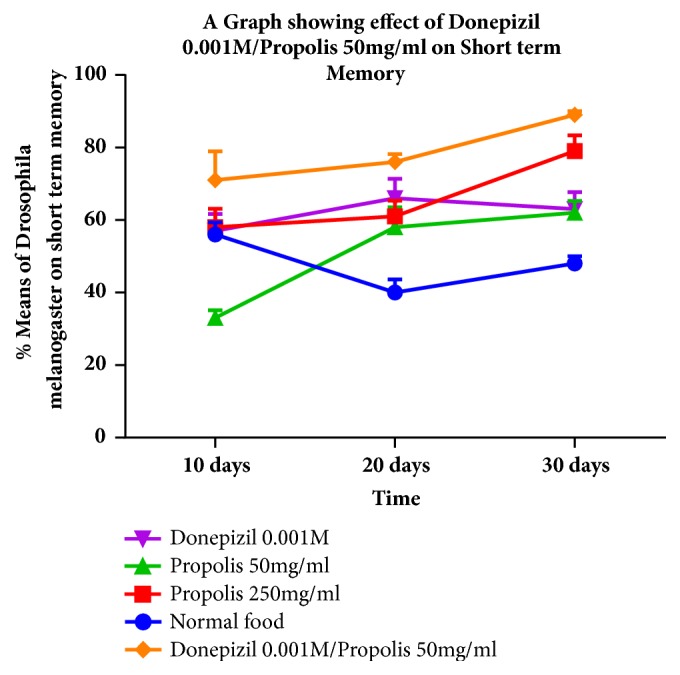
A graph showing the additive effect of the use of propolis with donepezil in the improvement of short term memory.

Propolis showed a dose dependent improvement in short-term memory with increase in number of days with the better results being observed with a higher dosage of 250 mg/ml of propolis (Figure 1).

**Figure 2:**
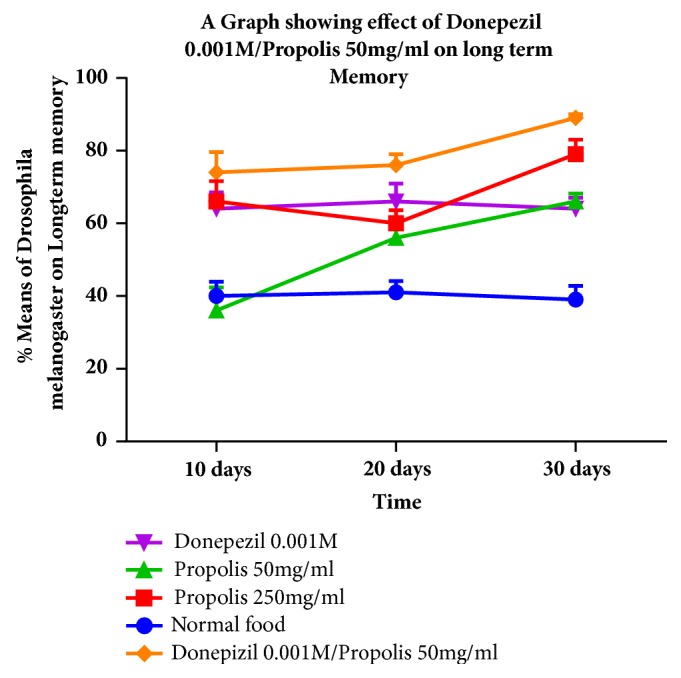
A graph showing the improvement in long term memory after the administration of donepezil 0.001/propolis 50 mg/ml of food for 30 days in wild type* Drosophila melanogaster *model.

There was a statistically significant improvement in the long-term memory observed up to values of 89.00%. This improvement was statistically significant when compared with the donepezil 0.001M group (Figure 2).

Propolis showed a dose dependent improvement in long-term memory. All the groups however registered a net improvement when compared with the wild type* Drosophila melanogaster *that was not administered either donepezil or propolis.

## 4. Discussion

Multiple aspects have been used to assess cognition including memory formation in* Drosophila* which include olfactory, courtship, appetitive, visual, and place memory. In this study, we focused on gustatory system to imprint the memory [23, 24].

The findings of this study revealed that the administration of propolis alongside donepezil which is a universally used acetylcholinesterase inhibitors a psychopharmaceutical in the treatment of Alzheimer's disease had a synergistic effect on cognition improvement in wild type* Drosophila melanogaster *models [25, 26]. Administration of the combination caused a much greater improvement in the short-term memory and in long-term memory than monotherapy of either. The cognition in our experiment was in association with aversive stimulus and phototaxis. We assessed the temporal aspect of cognition pattern in* Drosophila* which is categorized as either short- or long-term memory.* Drosophila melanogaster *naturally exhibit phototaxis and relatively high abilities to associate this with any aversive stimulus and this and other methods have been a cost effective means to study cognition in* Drosophila melanogaster *[23, 27].

Studies have shown that cognition in all animal species including humans decreases with increase in age [28, 29]. Most of the memory impairment during aging is believed to be a consequence of decline in neuronal function in cholinergic neurons and increase in neurodegeneration commonly associated with the hippocampus and its reduction in size [19, 30, 31]. These are all results of accumulation of oxidative damage and reduction of antioxidant defense system which play a key role in functional senescence by reducing the neuronal connections within the mushroom bodies in* Drosophila melanogaster* [32]. A study has shown that donepezil, when used in normal patients undergoing memory impairment disorders due to old age, in the long run would worsen memory impairment [26]. However, how the worsening in cognition came about was not clearly defined.

The group donepezil 0.001M/propolis 50 mg/ml that posed greater cognition improvement could be as a result of antioxidant effect of propolis. The present study is in agreement with the existing antioxidant effect of propolis; thus, it suggests that the mode of action of propolis in the improvement of cognition in normal individuals on donepezil treatment is based on its antioxidant effect. Our future studies would elucidate the phytochemical analysis of Ugandan propolis, isolate its bioactive components, and assess their effects on cognition using Alzheimer's disease model of* Drosophila melanogaster*.

## Figures and Tables

**Table 1:** 

**Group**	**Category**	**Treatment**	**Number of flies used**
Group 1	Wild type *Drosophila*	Standard commercial food With no additive	10 fruit flies

Group 2	Wild type *Drosophila*	Standard commercial food With 10mL of 0.00001M donepezil	10 fruit flies

Group 3	Wild type *Drosophila*	50mg/mL of propolis in food	10 fruit flies

Group 4	Wild type *Drosophila*	250mg/mL of propolis in food	10 fruit flies

Group 5	Wild type *Drosophila*	50mg of propolis+10mL of 0.00001M of donepizil	10 fruit flies

## Data Availability

The results in this article are entirely theoretical and analytical. The main steps of the demonstrations for each result are clearly reported in the text and the article is fully consistent without the support of any additional data.
